# Review: Allelochemicals as multi‐kingdom plant defence compounds: towards an integrated approach

**DOI:** 10.1002/ps.6076

**Published:** 2020-09-23

**Authors:** Darwin T Hickman, Amanda Rasmussen, Karl Ritz, Michael A Birkett, Paul Neve

**Affiliations:** ^1^ Rothamsted Research, Harpenden Hertfordshire UK; ^2^ University of Nottingham, Sutton Bonington Leicestershire UK

**Keywords:** allelopathy, plant defence, multi‐kingdom, secondary metabolites, fitness

## Abstract

The capability of synthetic pesticides to manage weeds, insect pests and pathogens in crops has diminished due to evolved resistance. Sustainable management is thus becoming more challenging. Novel solutions are needed and, given the ubiquity of biologically active secondary metabolites in nature, such compounds require further exploration as leads for novel crop protection chemistry. Despite improving understanding of allelochemicals, particularly in terms of their potential for use in weed control, their interactions with multiple biotic kingdoms have to date largely been examined in individual compounds and not as a recurrent phenomenon. Here, multi‐kingdom effects in allelochemicals are introduced by defining effects on various organisms, before exploring current understanding of the inducibility and possible ecological roles of these compounds with regard to the evolutionary arms race and dose–response relationships. Allelochemicals with functional benefits in multiple aspects of plant defence are described. Gathering these isolated areas of science under the unified umbrella of multi‐kingdom allelopathy encourages the development of naturally‐derived chemistries conferring defence to multiple discrete biotic stresses simultaneously, maximizing benefits in weed, insect and pathogen control, while potentially circumventing resistance. © 2020 The Authors. *Pest Management Science* published by John Wiley & Sons Ltd on behalf of Society of Chemical Industry.

## Graphical abstract

Plants have potential to synthesize metabolites for defence against multiple kingdoms; development of other plants, animal pests or microbial pathogens may all be inhibited by a single compound.

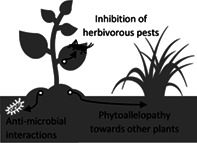

## 1 INTRODUCTION TO THE CONCEPT OF MULTI‐KINGDOM ALLELOPATHY

Allelopathy is defined in a broad sense as a phenomenon encompassing both the positive and negative effects of plants or microbes on other organisms by means of the chemicals, described as allelochemicals, which these species produce.^1^ This form of interference is distinct from resource competition, which is regulated by light, water or mineral nutrients.^2^ For the purposes of this review, we will consider allelopathy of plant species in a primarily detrimental context, as this provides most promise for crop protection and pest management.

The multi‐kingdom effects of some allelopathic plant secondary metabolites have long been acknowledged in definitions and discussions of allelopathy,^3^, ^4^ in spite of the original definition solely addressing plant–plant interactions.^5^ In the 1980s, multiple examples of compounds exhibiting allelopathy and toxicity to other organisms were defined,^6^ and the term ‘allelopathy’ was used in this context by the International Allelopathy Society in the 1990s.^1^ Other works have documented multiple ecological roles and applications for specific, individual plant‐derived secondary metabolites.^7^, ^8^, ^9^, ^10^ Works examining multi‐kingdom effects in allelopathic compounds nonetheless remain exceptional, with most literature focusing on the identification of inhibitory effects in novel natural compounds rather than their multi‐kingdom functions. This affects the scope of their applications for crop protection.

Allelochemicals are plant secondary metabolites, compounds considered nonessential for the direct development of cells, released into the environment *via* root exudation, leaching by precipitation, volatilization, or decomposition of plant tissues. Around 10 000 secondary metabolites have thus far been characterized from plant root exudates,^11^ complicating the isolation and elucidation of putative allelochemicals. There are few consistent terms for allelochemicals which may affect organisms of multiple kingdoms in the existing literature, and those that do exist serve different purposes to satisfy discussion of their individual disciplines. Considering such metabolites for multidisciplinary applications first requires clear definitions of these compounds.

In this review, the case is made that the existence of allelochemicals as defined above, with multiple ecological functions, necessitates the need for definitions that encompass both generic allelopathic interactions and more specific interactions with plants, animals and microbes. It is hereby suggested that ‘allelopathy’ is used in its wider definition in affecting multiple kingdoms as described previously,^1^, ^3^ and the terms ‘phytoallelopathy’, ‘zooallelopathy’ and ‘microbial allelopathy’ are used to describe specific interactions with plants, animals and microbes, respectively, in support of this. More detailed definitions of these terms as used throughout this review are provided in the text box. Having defined these interactions more clearly, it is now possible to describe the roles they could play in pest management.

Text box 1: Proposed definitions of allelopathy and associated terms regarding potential for multi‐kingdom applications.

**Table:** 

Allelopathy: The inhibition or stimulation of the growth or development of an organism through the biological action of secondary metabolites produced by plant species. These chemicals can be described as allelochemicals given this bioactivity, and will have effects on competition dynamics, and the stress tolerance of competitors.		
Phytoallelopathy: Allelopathy specifically towards another plant species, mediated by phytoallelochemicals.	Zooallelopathy: Allelopathy towards an animal species, typically an herbivore and most commonly observed in arthropods. This is mediated by zooallelochemicals.	Microbial allelopathy: Allelopathy towards a microbial species, such as a bacterium or fungus, mediated by antimicrobials, phytoalexins or phytoanticipins

Driven by the burgeoning issue of herbicide resistance in weeds,^1^ there is a growing need to develop more diverse and integrated weed management systems, to which phytoallelochemicals could contribute. As of 2020, herbicide resistance was reported in 262 species, to 167 herbicides, in 70 countries.^12^ Parallel to this, there is a growing cohort of insecticide‐resistant invertebrate species, with >600 species resistant to at least one insecticide mode of action in 2020,^13^ driving the desire for alternative approaches to their management. Fungicide resistance also is an issue, occurring in nine modes of action of fungicide by 2015.^14^ As a result, the recognition of multi‐kingdom allelochemicals which could potentially provide benefits against pesticide‐resistant organisms, and the development of control strategies which utilize these allelochemicals should be considered.

## 2 MULTI‐KINGDOM ALLELOCHEMICALS IN AN EVOLUTIONARY CONTEXT

### 2.1 Plant fitness and chemical defence

**Figure 1:**
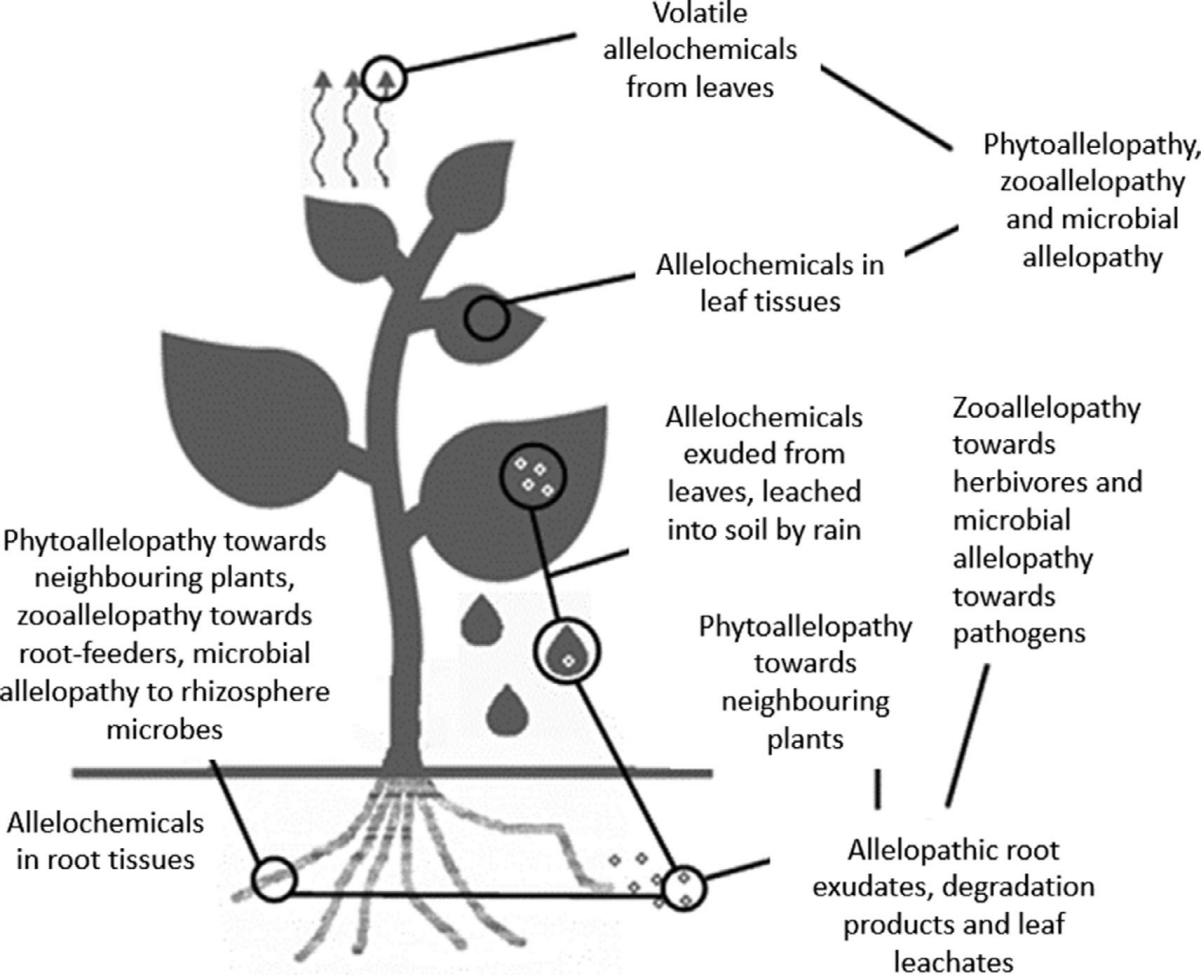
Antagonistic interactions between a plant and other species. The optimum defence allocation theory suggests that a single compound can confer resistance to multiple species, potentially of multiple kingdoms.

Plant productivity – and ultimately fitness – is not only impacted by resource competition with other plants, but also by herbivory, disease and stresses. Sessile plants cannot flee to avoid hostile organisms, so a key component of plant fitness is the ability to defend themselves by other means. Thus, evolution of generic defence mechanisms that maximize fitness would be of great benefit to plant species when faced with multiple stressor organisms. Indeed, it was posited that secondary metabolites provide general defence against multiple enemy organisms (Fig. 1).^6^ This assertion is connected to the optimal defence allocation theory, which suggests that allelochemicals are allocated to a greater extent where tissues are of greatest value, albeit encountering trade‐offs between growth, fecundity and defence.^15^ Allelopathy is thus linked to the ecological roles of these compounds through the vulnerability of different valuable tissues to different antagonistic organisms.

**Figure 2:**
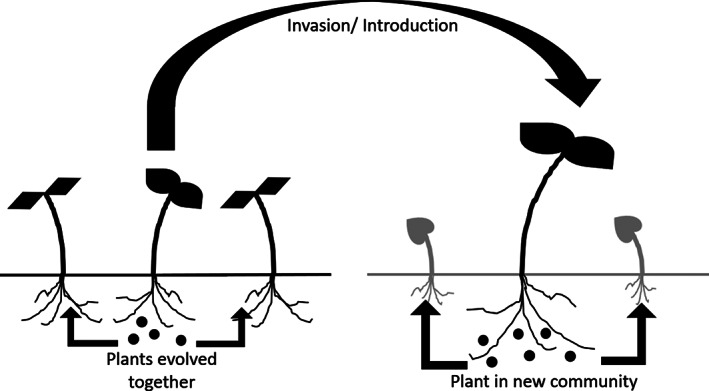
The Novel Weapons hypothesis with relation to allelochemical exudation and its inhibitory effects on unfamiliar competitors. Plants that have co‐evolved with a phytoallelopathic species have evolved a degree of tolerance; in a new community, as an invasive or introduced species, this allelochemical has stronger inhibitory effects against neighbours as they have not had opportunity for tolerance to evolve.

Plant defences also are affected by an evolutionary arms‐race, formalized by the ‘Red Queen’ hypothesis (Fig. 2). This hypothesis dictates that a species must constantly evolve adaptations to survive and thrive while faced with other species which are evolving in a similar way, effectively running as fast as it can to maintain its place, in the same manner as its namesake from *Through The Looking Glass*.^16^ Natural selection is therefore dynamic, and all species are constantly evolving to counter the defences of competitors, hosts or prey, to such an extent that the fitness of these organisms will decline unless natural selection facilitates the evolution of counter‐adaptations. It is thus ubiquitous across biological kingdoms, as it constitutes an element of maximizing ecological fitness.

### 2.2 Direct effects of phytoallelochemicals

**Figure 3:**
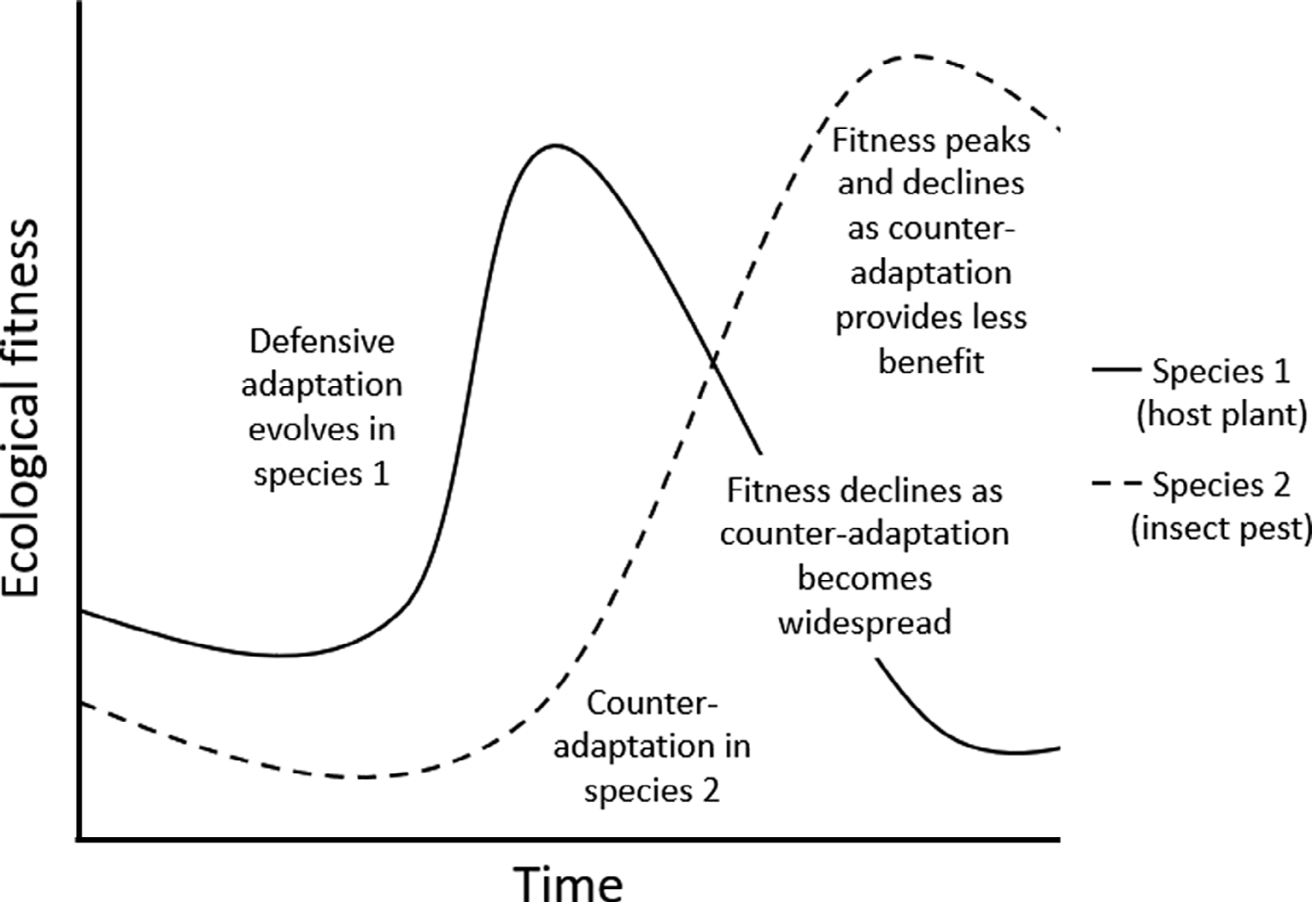
The ‘Red Queen’ hypothesis depicted in relation to how well a species is adapted to its environment, provided in this example in the case of a host plant (Species 1) and an insect pest (Species 2). As depicted, when Species 1 evolves a defensive adaptation, such as the exudation of a zooallelochemical, Species 2 declines in fitness, which constitutes a selection pressure for the evolution of a counter‐adaptation. Fitness increases in Species 2 and declines in Species 1 as this counter‐adaptation becomes widespread. This will either force a counter adaptation in Species 1 in order to ensure its survival.

The prevalence and possible ecological role of phytoallelopathy must first be examined in isolation to provide the basis for the wider phenomenon of multi‐kingdom effects. The ecological significance of phytoallelopathy is given weight by the study of invasive plants in natural ecosystems. Some invaders have the capacity to inhibit the development of would‐be local competitor plants through their phytoallelopathic interactions, which enable them to dominate invaded ecosystems. Examples include *Alliaria petiolata* and *Sonchus oleraceus*.^17^, ^18^ In both cases these interactions conform with the ‘novel weapons’ hypothesis (Fig. 3); in the case of *A. petiolata* this may be attributable to the action of glucosinolate compounds such as allyl isothiocyanate and benzyl isothiocyanate, whereas a number of potential allelochemicals have been identified in *S. oleraceus*. The phytoallelopathic potential and resulting disproportionate success of these species exists because resistance or tolerance has not evolved in this invaded ecosystem as would commonly be observed in the invader's native ecosystem.^19^ Phytoallelopathy in an agro‐ecological context, and the potential applications that this may have for agricultural benefit, have been reviewed extensively.^4^, ^20^, ^21^, ^22^

Sorghum species, and their phytoallelochemical sorgoleone, constitute an extensively studied and thoroughly reviewed example of phytoallelopathy at molecular, physiological and agroecological scales.^23^ The plant is known to have weed‐suppressive properties in the field,^23^ through the exudation of bioactive quantities of sorgoleone from root hairs.^24^ Sorgoleone is a potent phytoallelochemical, reducing *Digitalia sanguinalis* shoot growth by 50% at a dose of 10 μm, and reducing *Abutilon theophrasti* and *Echinochloa crus‐galli* development by the same degree at 200 μm.^25^ Multiple modes of action have been found in this compound, including the inhibition of photosynthetic and mitochondrial electron transport, the photosynthesis‐related enzyme *p*‐hydroxyphenylpyruvate dioxygenase (HPPD), and root H^+^‐ATPase activity required for water uptake.^23^

It should be noted that some plant secondary metabolites have indirect effects on the dynamics of resource competition. This may occur *via* stimulation of beneficial donor plant–microbe interactions, increasing donor competitive ability, or through phytoallelopathic effects, as reduced growth vigour in target plants culminates in reduced competitive ability. *Carduus nutans* root exudates, for instance, appear to be particularly inhibitory to legume species, starving soil of nitrogen over time and creating conditions to which the plant is comparatively tolerant.^26^ These effects may be attributable to the alkatetraene, aplotaxene.^27^ It is for this reason that some claim a separation of resource competition from phytoallelopathy to be unrealistic in an ecological context.^26^ It has been hypothesized that phytoallelopathy has evolved in reaction to intense resource competition to the detriment of the phytoallelopathic species.^28^ Phytoallelopathy and resource competition may thus be components of a complex web of rhizosphere‐based interactions involving nutrient availability (governing resource competition), exudation of secondary metabolites (including phytoallelochemicals) and soil microbial communities.^29^

### 2.3 Recognition and induction of allelochemical production

Allelopathic interactions in plants are likely to be influenced by recognition mechanisms, proposed to be mediated by chemical signalling in plant–plant interactions. The fitness benefit of phytoallelochemical exudation is optimized by inducibility,^30^ and as such the recognition of other plant species may constitute an important factor in phytoallelopathic behaviour. Such recognition can be influenced by both volatile aboveground and root‐secreted belowground stress‐related metabolites and proteins which appear to indicate the relatedness of a neighbour. There is growing evidence that allelochemical synthesis or exudation is elevated in response to recognition of neighbouring, competing plant species, a process that has been described as ‘allelobiosis’.^31^ The presence of root exudates from a number of weeds, specifically *Abutilon theophrasti*, *Aegilops tauschii*, *Amaranthus retroflexus* and *Digitaria sanguinalis*, all stimulated the accumulation of phytoallelochemicals in wheat.^31^ Bioassay of a wider variety of weed species indicated that phytoallelochemical accumulation in wheat varies depending on the identity of the competing species.^32^ This indicates that crop‐weed recognition is species‐specific, mediated by a wide range of diverse, and currently undefined, signalling compounds.

In phytoallelopathic plants, recognition interactions with competitive neighbours may be facilitated by phenotype matching: the ability of a plant to distinguish related individuals compared to those from other populations or species through chemical signatures.^33^ In parallel to another biotic kingdom, microbes contain recognition alleles, genes controlling the cues mediating recognition interactions, and therefore interact in a comparable manner in terms of recognition.^33^ Recognition interactions in plants, the compounds and systems involved, and how these influence phytoallelopathic mechanisms, are poorly understood, and require further elucidation. Competition stress and other environmental stress factors also are likely to influence allelopathy inducibility,^30^ but should be further examined to provide greater understanding.

There also is, conversely, evidence of allelochemical multi‐kingdom function in the induction of allelochemical synthesis; some allelochemicals accumulate *in planta* at atypically high levels when under pressure from herbivores, pathogens or both. For example, tissue disruption or wounding by the aphid *Rhopalosiphum padi* and the northern blight fungus *Setosphaeria turtica* stimulated allelochemical accumulation in maize.^34^ Likewise, feeding of *Psylliodes chrysocephala* on oilseed rape promotes the accumulation of multiple glucosinolates.^35^ This group of secondary metabolites is recognized for their phytoallelopathic potential.^36^ Thus, it is apparent that plants both recognize and react to multiple biotic stresses in a manner comparable to other organisms. Additionally, these inducible allelopathic mechanisms appear to have some consistency between multiple kingdoms of hostile organisms. It is thus logical that the compounds involved in these mechanisms have potential for multi‐kingdom effects.

### 2.4 Allelochemical allocation and fitness consequences

The theory of multi‐kingdom functionality in allelochemicals is dependent on ecologically rational allocation *in planta*. It is a reasonable extension of the optimal defence allocation theory that the distribution of a compound within a plant may be indicative of its fitness benefits.^15^ For example, benzoxazinoids, widely known as cereal phytoallelochemicals, are found at greater levels in wheat and rye roots than other tissues of these plants.^37^ Relative concentrations vary between wheat cultivars, however, and are greatest within a few days of germination, diminishing greatly as the plant develops.^38^ Glucosinolates and their isothiocyanate breakdown products, believed to be the primary allelochemicals in brassicaceous species, also accumulate at greater levels in roots.^39^ One could thus suggest that root exudate phytoallelopathy or microbial allelopathy to the rhizospheric community are the primary factors driving their selection. This can be disproven, at least in crop species such as wheat, which have undergone selection under unnatural conditions, by variability in phytoallelochemical exudation. Benzoxazinoid exudation was only detectable in 11 of 57 wheat cultivars despite all containing high concentrations within root tissues.^40^ It may thus be that allelochemical accumulation in root tissues provides the additional functional benefit of defence against root‐feeding herbivores such as the nematode *Pratylenchus neglectus*.^41^ Alternatively, the presence of high concentrations of allelochemicals in roots may be indicative of sequestration in root vacuoles, as has been reported with benzoxazinoids.^42^ This may prevent *in planta* autotoxic interactions which are harmful to vital plant tissues, rather than providing a direct fitness benefit. The apparent necessity of synthesizing and sequestering these compounds constitutes a fitness cost, which is likely to be overcome by a combination of benefits that confer a net competitive advantage.

Putative allelochemicals also can be found in high concentrations in aboveground tissues. This is particularly common in young tissues, which are of greater value to the plant due to their active growth, and thus allelochemical accumulation would appear to provide greater functional benefit as a feeding deterrent.^15^ This is the case in *Artemisia annua*, where artemisinin accumulates in flowers and buds, and is exuded from glandular trichomes on the surface of leaves and stems.^43^ Artemisinin is a potent phytoallelochemical, inhibiting the development of lettuce, as well as the weeds *Amaranthus retroflexus* and *Portulaca oleracea* at a concentration of 33 μm.^44^ There is evidence that artemisinin also is zooallelopathic to multiple arthropod species, indicating an additive functional benefit to this compound in relief of insect herbivory pressure. The beetle *Epilachna paenulata* and the armyworm *Spodoptera eridania* both suffered significant mortality when fed on pumpkin leaves treated with a dose of 1.5 mg cm^−2^ of artemisinin.^29^ One would thus assume zooallelopathy to be the primary fitness benefit conferred by this allocation. Even then, artemisinin may provide phytoallelopathic benefits in nature through leaching from the leaf surface by rainwater. Such an effect would be enabled by its relatively long half‐life in soil – around 30 days – ensuring that it would persist sufficiently for uptake by surrounding plant competitors.^43^ The influence of allelochemical persistence on their fitness benefits is further discussed later in this review.

In summary, the major benefit of allelochemical synthesis is likely to be defence against multiple hostile organisms, as would be suggested from the phenomenon of multi‐kingdom functionality. The resources required to produce such compounds and their tendency towards autotoxicity are major costs. Both appear to be minimized by the inducibility of synthesis in response to stress, and their tissue localization. The development of tolerance by a plant to the allelochemicals exuded into the environment is another potential adaptation to minimize fitness costs, as will be discussed in section 2.8.

### 2.5 Autotoxicity as a fitness cost

A further element in the discussion of multi‐kingdom allelochemicals is the existence and potential ecological role of autotoxicity, which disproves the specificity of these compounds to putative antagonistic species. Indeed, it should not be taken for granted that phytoallelopathic species are tolerant or resistant to their allelochemicals, and must thus still overcome autotoxicity in these compounds. Some of these compounds appear to have a degree of specificity in terms of their phytoallelopathy, but others do not, so their producers reduce associated fitness costs through inducibility, localization and tolerance. Multiple plant species still exhibit a degree of autotoxicity, including wheat^45^ and *Sonchus olearaceus*.^17^ These species produce root exudates with both phytoallelopathic and autotoxic potential. Few studies have successfully elucidated autotoxic compounds, but where they have, interspecific phytoallelochemicals are among such compounds; In alfalfa, for instance, the compounds of greatest effect were coumarins, *trans*‐cinnamic acid and *o*‐coumaric acid.^8^, ^46^ This would suggest that some phytoallelochemicals may also act as autotoxins, although their effects are likely to have evolved to confer some fitness benefit to their target. Artemisinin also represents an autotoxic phytoallelochemical, a dose of 33 μm significantly reducing *Artemisia annua* germination and seedling development.^44^ In this case, autotoxicity is avoided by localization, protecting the producing cell's cytoplasm through restricting the compound to the subcuticular space of the glandular trichomes while *in planta*.^43^

The reasons for the evolution of autotoxicity are not clear, although explanations have been posited which rationalize the phenomenon in spite of the existence of the aforementioned adaptations which would seemingly prevent it. A commonly suggested hypothesis is that of biochemical recognition, which postulates that intraspecific inhibition of germination provides selective advantages for population fitness in the avoidance of intense intraspecific competition, favouring later germination and establishment when conditions are more suitable.^47^ This can be compared to phytoalexin‐regulated hypersensitive cell death to contain pathogenic infection, one example being in response to resveratrol in pathogen‐infected grape plants.^48^ Another hypothesis concerning the existence of autotoxicity in an ecological setting is more simplistic; it is possible that there is an unavoidable fitness cost associated with the production and maintenance of more effective defences against other, more pressing stresses. The compounds involved must be conferring considerable fitness benefits in this case, which may be explained by their multi‐kingdom potential.

### 2.6 Hormesis and the dose question

A possible alternative explanation for the existence of autotoxicity is that it is an undesired fitness cost relating to the promotion of hormesis: the stimulation of growth at low concentrations by compounds that are known or suspected to be detrimental at higher concentrations. Hormesis specifically occurs at around one‐tenth of an effective inhibitory dose.^49^ Several reasons for hormesis of autotoxins have been discussed, including the theory that exudation of these compounds is intended to stimulate, rather than inhibit, further growth of the species.^50^ In the case of hormesis, inhibitory effects would occur as a consequence of unnaturally high plant density, such as in a planted monoculture field. Alternatively, exudation may be overstimulated to the detriment of the producing species by other stress factors, including the presence of competitors, underpinned by the recognition interactions described earlier. The occurrence of autotoxicity would therefore be a consequence of the dose‐dependency of phytoallelochemicals. Hormesis was reported in some wheat lines,^45^ as well as in a number of cases where pure phytoallelochemicals were applied to target species.^49^

Hormesis is known to occur additionally in synthetic herbicides such as glyphosate and bromoxynil.^49^ It also appears to occur in inhibition of arthropods by zooallelochemicals, as has been observed in *Azadirachta indica*‐derived azadirachtin applied to the bean weevil, *Zabrotes subfasciatus*.^51^ The phenomenon manifests itself as a trade‐off in this case, however, with the effect of increasing fecundity but reducing longevity in an apparent case of r‐selection.^51^

Hormesis and autotoxicity exemplify two extreme outcomes in the governance of the ‘Paracelsus axiom’ over allelochemical interactions. This is the theory that toxicity is only ever determined by dose, and by extension, all compounds can exhibit stimulatory and inhibitory interactions towards an organism at the correct dose.^49^ In the case of hormesis, allelopathic behaviour is not likely to be detrimental; indeed it would be of ecological and evolutionary benefit for a plant to evolve the synthesis of a compound stimulatory to growth of kin and inhibitory to competitors at low concentrations, allowing their benefit from plentiful resources in their environs while inhibiting competitors, but which became autotoxic at higher concentrations where seed germination is inhibited at times of intense intraspecific competition.

### 2.7 Allelochemical persistence in the environment

The environmental fate of allelochemicals in soil also is a noteworthy factor in their evolution and activity towards multiple kingdoms. A degree of persistence is necessary for a compound to induce phytoallelopathy or microbial allelopathy in nature, albeit not to the degree that resistance would evolve. Many phytoallelochemicals are degraded by microbial action, such as simple phenolic acids, benzoxazinoids, juglone, quercetin, rutin and *meta*‐tyrosine,^52^ some of which exhibit multi‐kingdom effects, which will be reviewed in the next section. The effect of degradation on phytoallelopathic bioactivity can be profound. For example, of nine weed species reported in one study to have phytoallelopathic root exudates, only one, *Ageratum conyzoides*, maintained its bioactivity in unsterilized soil.^53^ For this reason, many bioassays investigating the potency of phytoallelochemicals in artificial conditions such as sterile soil could overestimate their effects.^52^, ^53^ Difficulty in proving in‐field phytoallelopathy gives credence to the perspective that studies in these artificial conditions are ecologically irrelevant.^54^ Rather, the ideal study of a putative allelopathic species or compound should begin with a simplified laboratory model which is necessary to elucidate its effects and modes of action. This should be followed with assays in more ecologically relevant conditions, culminating in in‐field bioassays to ensure their applicability.

The benzoxazinoid allelochemicals DIMBOA (2,4‐dihydroxy‐7‐methoxy‐1,4‐benzoxazin‐3‐one) and DIBOA (2,4‐dihydroxy‐1,4‐benzoxazin‐3‐one) degrade quickly in unsterilized soil, with half‐lives of around 24 h^45^ and 43 h,^55^ respectively. Rapid degradation of allelochemicals sometimes can result in more persistent compounds with greater bioactivity, and therefore may be an ecologically rational strategy in this case. For example, phytotoxic APO (2‐amino‐phenoxazin‐3‐one), a degradation product of DIBOA, persists for ≤90 days in biologically active soil, part of the reason for its acknowledgement by some as an important component in cereal phytoallelopathy.^55^

### 2.8 Resistance and tolerance to allelochemicals

There is a propensity for resistance to allelochemical compounds to evolve, in much the same manner as resistance to synthetic pesticides. It is for this reason that multi‐kingdom effects are not universal at uniform concentrations. Evolution of resistance occurs as a natural ebb‐and‐flow of the evolutionary arms race in a natural ecosystem, but by extension, evidence which will be discussed in this section suggests that such developments could facilitate the use of allelochemicals as naturally‐inspired crop protection compounds. Indeed, evolution of tolerance or resistance by a target species in its natural setting may be the primary reason for limitations in the universality of such compounds.

Multiple fungal wheat pathogens, including several *Fusarium* species,^37^ and several plant species, have evolved the ability to detoxify benzoxazinoids, for instance.^42^ Likewise, the presence of low concentrations of glucosinolate compounds from *Alliaria petiolata*, as a result of partial degradation by the native rhizosphere community, is linked to eventual resistance of these microbes to these compounds.^56^ Insect herbivores can also evolve tolerance to secondary plant metabolites, circumventing zooallelopathic defences through counter‐resistance evolved in the manner suggested by the ‘Red Queen’ hypothesis. This is particularly apparent where host resistance is encoded by just one gene, with selectively bred lettuce resistant to the aphid *Pemphigus bursarius* for just ten years before the aphid evolved counter‐resistance.^57^ Similar dynamics are apparent in various lepidoptera that evolved mechanisms to glycosylate DIMBOA back to its nontoxic storage form.^58^ The DIBOA degradation product BOA (benzoxazin‐2‐one) can furthermore be detoxified by glutathione transferase (GST) and cytochrome P450 monooxygenase (CYP‐P450) activity in *Arabidopsis*.^59^ Thus, from an ecological perspective, the benefit of novel weapons will ultimately be overcome by counter‐selection. The vulnerability of native ecosystems to the allelochemicals of an invading plant species will be overcome by the evolving resistance of native species in time, but this first requires the invader to become dominant and disrupt the ecosystem, thereby creating an intense selection pressure.

There also appears to be further association in the form of cross‐resistance, as insect pests of allelopathic herbaceous species have a greater likelihood of evolving resistance to synthetic pesticides. A recent example of this can be found in the cotton bollworm (*Helicoverpa armigera*), which exhibited reduced larval sensitivity to the synthetic insecticide methomyl when fed with a number of allelochemicals including coumarin and DIMBOA. This metabolic cross‐resistance was correlated with elevated activity of both GSTs and CYP‐P450s, which often confer resistance.^60^ This is connected with the theory of pre‐adaptation, that the mechanisms to detoxify zooallelochemicals of insect pests may incidentally provide a degree of pre‐adaptation to synthetic insecticides.^61^ In parallel to the changing efficacy of synthetic herbicides, control of resistant insect species is becoming more difficult due to an over‐reliance on these insecticides. The dynamics of pre‐adaptation need to be further explored in order to facilitate more effective application of allelochemical‐derived biocides.

## 3 EXAMPLES OF ALLELOCHEMICAL MULTI‐KINGDOM FUNCTIONALITY

An integrated approach which takes account of the multi‐kingdom behaviour of allelochemicals could optimize benefit in terms of crop yield. It is important to consider individual compounds within this multi‐kingdom framework. To this end, the examples of benzoxazinoids, *meta*‐tyrosine and juglone, are presented as multi‐kingdom allelochemicals that give credence to this recurring concept. Such examples are not exhaustive, and also include momilactones in rice, which are both phytoallelochemicals^62^ and phytoalexins,^63^ and parthenin from *Parthenium hysterophorus*, which is both phyto‐^64^ and zooallelopathic.^65^ Table 1 summarizes the multi‐kingdom effects presented in this section.

**Table 1:** Summary of multi‐kingdom effects in allelochemicals discussed in Section 3

Allelochemical	Plant producer	Phytoallelopathy	Zooallelopathy	Microbial allelopathy
Benzoxazinoids	Various^66^	*Sinapis alba*, *Lolium rigidum*, *Avena fatua* ^66^, ^67^	*Ostrinia nubilalis*, *Diuraphis noxia*, *Meloidogyne incognita* ^68^, ^69^, ^70^	Various^71^, ^72^
*Meta*‐tyrosine	*Festuca rubra*	*Digitaria sanguinalis*, *Trifolium repens*, *Taraxacum officinale* ^73^	*Coptotermes formosanus* ^74^	*Bacillus* spp.^75^
Juglone	*Juglans nigra* ^9^	Various^76^	*Callosamia promethea* ^77^	Various^78^

### 3.1 Benzoxazinoids

Benzoxazinoids are a family of cyclic hydroxamic acids synthesized by a range of plant species, and long‐studied for their biological activity. Benzoxazinoids are widespread in nature, occurring in Acanthaceae, Ranunculaceae, Scrophulariaceae and Poaceae,^66^ including wheat, rye, barley and maize.^37^ The two benzoxazinoids most commonly attributed to conferring wheat allelopathy are DIMBOA and DIBOA, and their breakdown products.^79^ DIBOA was discovered in 1959, and DIMBOA in 1962, although their phytoallelopathic potential was not discerned until the 1990s.^79^ These compounds degrade to MBOA (6‐Methoxy‐2‐benzoxazolinone) and BOA, respectively, which then degrade further into AMPO (2‐amino‐7‐methoxy‐phenoxazin‐3‐one) and APO, respectively, as has been reviewed previously.^80^

All of these compounds have been tested on multiple target species and considered as putative phytoallelochemicals.^67^, ^81^, ^82^ Elevated benzoxazinoid exudation by multiple cereal species correlates with the suppression of *Sinapis alba* development, indicative of phytoallelopathy.^66^ A 500 μm dose of DIMBOA is sufficient to inhibit root length in *Avena fatua* by ≈70% and *Lolium rigidum* by ≈55%, compared to controls.^67^ DIMBOA isolated from wheat root exudates reduced dry weight of *Alopecurus aequalis* by ≈20%.^83^ Because a similar biomass reduction (21%) in test plant species was caused by crude wheat root exudates,^84^ DIMBOA would appear to be the primary phytoallelochemical exuded by the species.^83^

DIBOA is likewise phytoallelopathic to DIMBOA, also inhibiting *Lolium rigidum* at a dose of 500 μm and *Avena fatua* at 100 μm.^67^ When DIBOA was applied axenically to oat and broad bean plants, H^+^ATPase activity in roots was reduced.^85^ This is likely to be related to the electrophilicity of DIBOA, its attraction to electrons and electron‐dense molecules.^7^ Therefore, benzoxazinoids appear to limit supply of adenosine triphosphate (ATP) by inhibiting electron transport, hindering the mechanisms by which cells release energy.

Conversely, this is only one of many suggested modes of action posited for benzoxazinoid allelochemicals. Treatment with these compounds has led to a number of effects, including reduced activity of other enzymes such as papain, *α*‐chymotrypsin and GSTs.^86^ The mode of action has not been conclusively identified for DIMBOA or DIBOA, or their respective degradation products,^87^ and has been elucidated only in APO and AMPO.^88^ These compounds bind to and inhibit the action of highly‐conserved histone deacetylase (HDAC) enzymes, which are necessary for amino acid transcription and therefore cell development.^88^ Such effects occur at concentrations as low as 3.25 μm, sufficient for physiological relevance.^87^ This explains the notable allelopathic potency of APO in particular, being a much more potent phytoallelochemical than DIMBOA or DIBOA.^81^

Some benzoxazinoids confer zooallelopathy against invertebrate herbivores, known long before their phytoallelopathic potential was discovered. DIMBOA is inhibitory to larval development in the European corn borer *Ostrinia nubialis*, translating to a 25% mortality rate at a concentration of ≈1.5 mm kg^−1^ in no‐choice diet assays.^68^ Likewise, DIBOA in wild barley species negatively impacted development of the aphid *Diuraphis noxia*,^69^ and when exuded from rye, also inhibited egg development of the nematode *Meloidogyne incognita*.^70^ This suggests that both DIMBOA and DIBOA are broadly toxic to invertebrate species. This assertion seems reasonable given that higher benzoxazinoid content in wheat leaves correlated with enhanced resistance to various aphid species at naturally relevant concentrations of ≈3 mm kg^−1^ fresh weight.^89^

Benzoxazinoids additionally have well‐documented antimicrobial potential. BOA, the primary degradation product of DIBOA, was first discovered as an antifungal agent against pathogenic *Fusarium* species.^71^ Moreover, multiple bacteria and yeasts are sensitive to DIMBOA, DIBOA and BOA at concentrations typically <3 mm,^72^ suggesting that this family of compounds have applications as broad‐spectrum antimicrobials. As benzoxazinoids have been suggested to inhibit ATP synthesis, central to all life excepting viruses, it is logical that they would be toxic to multiple taxa of plants, animals and microbes.

The examples offered here form a strong case for phytoallelochemicals having applications in other areas of plant defence, and strongly indicates that benzoxazinoids offer leads for potential development of pesticides with multiple applications. This is further corroborated by the considerable research into the various functions of these compounds, as well as the relationship that chemical structure has on these functions, which has already been reviewed in great detail elsewhere.^7^, ^90^

### 3.2 *Meta*‐tyrosine

Grasses such as *Festuca rubra* exude *meta*‐tyrosine, the active compound inhibiting root growth in bioassays of crude root exudates from the species. *Meta*‐tyrosine inhibited a number of species including weeds such as *Digitaria sanguinalis*, *Trifolium repens* and *Taraxacum officinale*.^73^ The compound also inhibited *Arabidopsis* root length by 50% at a concentration of 25 μm – a potent phytoallelopathic effect.^73^ *Arabidopsis* root tip browning was observed in the phytoallelopathic activity of *m*‐tyrosine, indicative of cell necrosis.^91^ Leaf necrosis has also been reported in *m*‐tyrosine‐treated *Arabidopsis* at a concentration of 40 μm.^92^ Nonprotein amino acids are thought to have phytotoxic properties through their substitution of protein amino acids during translation, modifying protein folding as a result.^73^ This mode of action has recently been verified for *m*‐tyrosine, which is specifically misincorporated in place of phenylalanine.^92^

Despite its apparent specificity to plant proteins in terms of their mode of action,^73^ there is evidence of allelopathy towards other organisms by *m‐*tyrosine. A higher concentration than that required to confer phytoallelopathy (50 mm) results in antifeedant and toxic effects on the termite *Coptotermes formosanus*.^74^ The development and sporulation of multiple *Bacillus* bacterial species was inhibited by 500 μm of *m*‐tyrosine.^75^

It is likely that *m*‐tyrosine is capable of providing multi‐kingdom toxicity. This is in spite of an apparent specificity to plant proteins which would explain evidence that zooallelopathy may be an unrealistic expectation at natural concentrations. Thus it may be that the observed wider allelopathic effects could potentially be conferred by other, yet undiscovered, mechanisms.

### 3.3 Juglone

The phytoallelopathy of juglone, a naphthoquinone produced by walnut trees, particularly *Juglans nigra*, was discovered in the late 1800s.^9^ The inhibitory effects of juglone on other plant species have been widely explored and documented.^9^ For example, assay of the effects of juglone on 16 herbaceous and woody plant species both on blotter paper and in soil, found dry weight of five species to be significantly inhibited by a concentration of 10 μm, whereas a further ten species were affected at a concentration of 100 μm.^76^ The dry weight of *Lemna minor* also was significantly reduced by a 10 μm dose of juglone, with a reduction in net photosynthetic activity seemingly related to mitochondrial disruption.^93^ Although a number of modes of action have been theorized and none confirmed for juglone, inhibition of corn and soybean development at similar concentrations were associated with mitochondrial inhibition in root cells through the reduction of H^+^ATPase activity, and the disruption of plasma membrane function.^94^ It is therefore apparent that juglone is phytoallelopathic to a wide range of plant species, as past reviews have discussed.^95^

The growth rate of the promethea silkmoth (*Callosamia promethea*) was reduced 3.6‐fold when fed on leaves treated with 0.05% juglone (w/w), similar to the concentration in black walnut leaves.^77^ It would therefore seem apparent that the compound has additional zooallelopathic potential. Juglone also exhibits a degree of microbial allelopathy to a wide range of plant pathogens, which were significantly inhibited at a concentration of 75 μm.^78^ Fungal species in particular seemed highly sensitive to the compound, to the extent that effects of juglone are comparable to those of some commercial antifungal agents.^78^ It would therefore appear that juglone exhibits a degree of multi‐kingdom functionality, the full range of which is apparent from extensive review of its biological effects.^9^

## 4 WHAT DOES MULTI‐KINGDOM FUNCTIONALITY MEAN FOR CROP PROTECTION?

### 4.1 Potential applications of multi‐kingdom allelochemicals

As the examples provided throughout this review indicate, a number of crop species are involved in multi‐kingdom allelopathic interactions. Bringing such multi‐kingdom effects to application for the benefit of agroecosystems first requires consideration of factors influencing in‐field crop allelopathy, and broader ecological impacts, both of which have been reviewed by a number of important works.^4^, ^22^, ^96^ Ancestor varieties of domesticated crops often appear to be more potent producers of allelochemicals, so there is interest in assessing and re‐introducing this material into breeding programmes to augment their natural defences.^97^ Few such breeding programmes have been explored, even solely for weed suppression. The prime example in this case is rice, where weed suppression related to competitive and phytoallelopathic potential have been widely characterized.^98^

Such multi‐kingdom allelochemicals would also provide leads for the development of future pesticides. They often are multi‐target site inhibitors,^99^ and may thus provide defence against multiple biotic threats as a result. Prioritizing the development of such multi‐site inhibitors has recently been advocated given the greater difficulty of evolving resistance against multiple targets.^100^ It is hereby suggested by extension that multi‐kingdom functionality may be an added, ecologically rational benefit, and provide a broader‐ranging basis for pesticide development and deployment in crop protection. From a practical perspective, developing naturally‐inspired biocides protective against multiple biotic pressures is economically and agronomically rational.

There are, by comparison, multiple examples of insecticides developed from zooallelochemicals.^101^ Examples include pyrethroids developed from the pyrethrins found in *Chrysanthemum* species, and insecticides derived from *Azadirachta indica*, which have been reviewed extensively.^102^ Even then, this is an underdeveloped tool in crop protection. More pertinently to this review, there are no records of allelochemicals which have inspired the development of multi‐kingdom pesticides, in spite of the examples of multi‐kingdom functionality posited throughout.

### 4.2 Barriers to development of natural product‐based pesticides

There are a number of contributory reasons for the underdevelopment of natural product‐based pesticides, particularly herbicides. A major caveat of harnessing phytoallelochemicals is their potential for nontarget effects. *Poecilus cupreus* larvae and *Folsomia candida* springtails are beneficial soil organisms detrimentally affected by these compounds.^103^ APO is also inhibitory to the growth and development of the water flea *Daphnia magna*, used as an indicator of aquatic pollution.^104^ It is of course a necessity to fully determine the full environmental impact of a new crop protection compound, which is not excused by the perceived environmentally benign nature of allelochemicals or allelochemical‐inspired formulations. High concentrations of allelochemicals may be required to elicit the desired inhibitory effects, moreover, as a result of some degree of tolerance. This issue can be minimized by the identification of a maximum relevant dose, be it in terms of how much can be synthesized while remaining economically viable, or in terms of the concentrations of these compounds occurring in the allelopathic plant. The correct dose is further necessitated by hormesis, as there becomes a concern that the incorrect dose could stimulate, rather than inhibit, the growth of a detrimental species.

The development of a breeding programme for phytoallelopathic potential is dependent on a huge amount of knowledge.^4^ The germplasm of a given species must be explored widely for phytotoxic potential, and this must be proven consistently on multiple relevant target species.^98^ Myriad (in some cases poorly understood) factors which can influence allelochemical synthesis and exudation, including the recognition interactions described in Section 2.3, as well as the influences of pest insects, pathogens and environmental factors; all of these must be understood for a breeding programme to succeed and provide agronomic benefit.^90^ Dynamics of allelochemical degradation in field soil must be characterized to ensure that there is not only no detriment to succeeding crops, but also that a focal compound persists sufficiently to have biological effects,^101^ which means that the active allelochemicals must therefore be identified.^4^, ^90^ Crops produced by a breeding programme need to maintain comparable yield to those currently commercialized, which must be extensively examined before release.^98^ There is therefore a large amount of interdisciplinary work attached to the development of a viable agronomic outcome, and this is increased significantly when multi‐kingdom effects are desired. It is for this reason that crop protection products based on allelopathy are rare, but not impossible to produce.

## 5 PERSPECTIVE

Given the number of existing examples of apparent phytoallelochemicals with antimicrobial or zooallelopathic properties, it is apparent that these compounds exhibit a degree of multi‐kingdom functionality. This must be a result of these defences co‐evolving to confer an overall net fitness benefit in natural habitats, likely to constitute tolerance to herbivores, plant competitors and soil microbes.

Therefore, it is acknowledged that phytoallelochemicals are a sub‐class of multi‐kingdom inhibitors, and all of these compounds are allelochemicals. It is unlikely that biosynthesis and release of currently‐recognized allelochemicals has evolved entirely as a result of the functional benefit of phytoallelopathy, given the distribution of a number of these compounds aboveground *in planta* and the dynamics associated with such allocation.

From a practical perspective, this means that allelochemical compounds, delivered as weed management tools either through enhanced production and delivery *in planta* via crop breeding or genetic engineering, or through the production of pesticide formulations using these chemicals as leads, may in fact have application in plant defence to multiple biotic stresses. Testing would be required, however, given that resistance, tolerance, or other factors may exist detrimental to the multi‐kingdom functionality of some allelochemicals. It remains highly likely that there exist other examples of previously researched phytoallelochemicals which have currently not been examined for multi‐kingdom effects, but which exhibit them.

Conversely, the area of phytoallelochemical discovery is currently hindered by its reliance on the demonstration of phytoallelopathy, a notoriously difficult phenomenon to demonstrate in isolation; it is hereby argued that it would benefit from greater consideration of compounds with proven allelopathic effects on herbivorous pests or microbial pathogens. The hope is that the identification and development of such multi‐kingdom inhibiting, naturally‐derived pesticides would delay the evolution of further resistance to existing synthetic chemistries while also providing effective new tools for weed, arthropod and pathogen management.

The future outlined here would be realized by the testing of potent allelochemicals with little documented evidence of multi‐kingdom functionality for this effect in problematic target species. The adoption of such a multidisciplinary outlook in informing the discovery of potential crop protection compounds has the potential to reduce the considerable time and economic cost required to bring new natural product formulations to market^105^ by reducing the likelihood of producing and testing ineffective compounds, thereby benefitting both consumers and industry.

## References

[1] Mallik AU and Inderjit S , Problems and prospects in the study of plant allelochemicals: a brief introduction, in Chemical Ecology of Plants: Allelopathy in Aquatic and Terrestrial Ecosystems, ed. by Mallik AU and Inderjit S Birkhäuser Verlag, Basel, pp. 1–5 (2002).

[2] Duke SO , Proving allelopathy in crop–weed interactions. Weed Sci 63:121–132 (2015).

[3] Whittaker RH and Feeny PP , Allelochemics: chemical interactions between species. Science 171:757–770 (1971). 5541160 10.1126/science.171.3973.757

[4] Putnam AR and Duke WB , Allelopathy in agroecosystems. Annu Rev Phytopathol 16:431–451 (1978).

[5] Molisch H , Der einfluss einer pflanz auf die andere– allelopathie. Gustav Fischer, Jena (1937).

[6] Wink M , Plant breeding: importance of plant secondary metabolites for protection against pathogens and herbivores. Theor Appl Genet 75:225–233 (1988).

[7] Wouters FC , Gershenzon J and Vassão DG , Benzoxazinoids: reactivity and modes of action of a versatile class of plant chemical defenses. J Braz Chem Soc 27:1379–1397 (2016).

[8] Siqueira JO , Hammerschmidt R and Nair MG , Significance of phenolic compounds in plant‐soil‐microbial systems. CRC Crit Rev Plant Sci 10:63–121 (1991).

[9] Strugstad MP and Despotovski S , A summary of extraction, synthesis, properties, and potential uses of juglone: a literature review. J Ecosyst Manag 13:1–16 (2012).

[10] Schandry N and Becker C , Allelopathic plants: models for studying plant–interkingdom interactions. Trends Plant Sci 25:176–185 (2020). 31837955 10.1016/j.tplants.2019.11.004

[11] Uren NC , Types, amounts, and possible functions of compounds released into the rhizosphere by soil‐grown plants, in The Rhizosphere: Biochemistry and Organic Substances at the Soil‐Plant Interface, ed. by Pinton R , Varanini Z and Nannipieri P Marcel Dekker, Inc., New York, NY, pp. 19–40 (2001).

[12] Heap I , International survey of herbicide resistant weeds, 2020 http://weedscience.org/Summary/Species.aspx?WeedID=6 [3 February 2020].

[13] Whalon ME , Mota‐Sanchez D , Hollingworth RM and Duynslager L , Arthropod pesticide resistance database, 2020 https://www.pesticideresistance.org/ [accessed 2 March 2020].

[14] Lucas JA , Hawkins NJ and Fraaije BA , The evolution of fungicide resistance, in Advances in Applied Microbiology, ed. by Sariaslani S and Gadd GM Elsevier Ltd., Amsterdam (2015). 10.1016/bs.aambs.2014.09.001 25596029

[15] McCall AC and Fordyce JA , Can optimal defence theory be used to predict the distribution of plant chemical defences? J Ecol 98:985–992 (2010).

[16] Benton MJ , The red queen and the court jester: species diversity and the role of biotic and abiotic factors through time. Science 323:728–732 (2009). 19197051 10.1126/science.1157719

[17] Gomaa NH , Hassan MO , Fahmy GM , González L , Hammouda O and Atteya AM , Allelopathic effects of *Sonchus oleraceus* L. on the germination and seedling growth of crop and weed species. Acta Bot Brasilica 28:408–416 (2014).

[18] Prati D and Bossdorf O , Allelopathic inhibition of germination by *Alliaria petiolata* (Brassicaceae). Am J Bot 91:285–288 (2004). 21653384 10.3732/ajb.91.2.285

[19] Callaway RM and Ridenour WM , Novel weapons: invasive success and the evolution of increased competitive ability. Front Ecol Environ 2:436–443 (2004).

[20] Qasem JR and Foy CL , Weed allelopathy, its ecological impacts and future prospects: a review. J Crop Prod 4:43–119 (2001).

[21] Jabran K , Mahajan G , Sardana V and Chauhan BS , Allelopathy for weed control in agricultural systems. Crop Prot 72:57–65 (2015).

[22] Weston LA and Duke SO , Weed and crop allelopathy. CRC Crit Rev Plant Sci 22:367–389 (2003).

[23] Weston LA , Alsaadawi IS and Baerson SR , Sorghum allelopathy‐from ecosystem to molecule. J Chem Ecol 39:142–153 (2013). 23393005 10.1007/s10886-013-0245-8

[24] Czarnota MA , Paul RN , Dayan FE , Nimbal CI and Weston LA , Mode of action, localization of production, chemical nature, and activity of sorgoleone: a potent PSII inhibitor in *Sorghum* spp. root exudates. Weed Technol 15:813–825 (2001).

[25] Nimbal CI , Pedersen JF , Yerkes CN , Weston LA and Weller SC , Phytotoxicity and distribution of sorgoleone in grain sorghum germplasm. J Agric Food Chem 44:1343–1347 (1996).

[26] Inderjit S and Callaway RM , Experimental designs for the study of allelopathy. Plant and Soil 256:1–11 (2003).

[27] Silva FML , Donega MA , Cerdeira AL , Corniani N , Velini ED , Cantrell CL *et al*, Roots of the invasive species *Carduus nutans* L. and *C. acanthoides* L. produce large amounts of aplotaxene, a possible allelochemical. J Chem Ecol 40:276–284 (2014). 24557607 10.1007/s10886-014-0390-8

[28] Williamson GB , Allelopathy, Koch's postulates, and the neck riddle, in Perspectives on Plant Competition, ed. by Grace JB and Tilman D Academic Press Inc., San Diego, CA, pp. 142–162 (1990).

[29] Maggi ME , Mangeaud A , Carpinella MC , Ferrayoli CG , Valladares GR and Palacios SM , Laboratory evaluation of *Artemisia annua* L. extract and artemisinin activity against *Epilachna paenulata* and *Spodoptera eridania* . J Chem Ecol 31:1527–1536 (2005). 16222790 10.1007/s10886-005-5795-y

[30] Uesugi A , Johnson R and Kessler A , Context‐dependent induction of allelopathy in plants under competition. Oikos 128:1492–1502 (2019).

[31] Li Y‐H , Xia Z‐C and Kong C‐H , Allelobiosis in the interference of allelopathic wheat with weeds. Pest Manag Sci 72:2146–2153 (2016). 26833449 10.1002/ps.4246

[32] Kong C‐H , Zhang S‐Z , Li Y‐H , Xia Z‐C , Yang X‐F , Meiners SJ *et al*, Plant neighbor detection and allelochemical response are driven by root‐secreted signaling chemicals. Nat Commun 9:3867 (2018). 30250243 10.1038/s41467-018-06429-1 PMC6155373

[33] Penn DG and Frommen JG , Kin recognition: an overview of conceptual issues, mechanisms and evolutionary theory, in Animal Behaviour: Evolution and Mechanisms, ed. by, ed. by Kappeler P Springer, Heidelberg, pp. 55–85 (2010).

[34] Ahmad S , Veyrat N , Gordon‐Weeks R , Zhang Y , Martin JL , Smart L *et al*, Benzoxazinoid metabolites regulate innate immunity against aphids and fungi in maize. Plant Physiol 157:317–327 (2011). 21730199 10.1104/pp.111.180224 PMC3165881

[35] Bartlet E , Kiddle G , Williams I and Wallsgrove R , Wound‐induced increases in the glucosinolate content of oilseed rape and their effect on subsequent herbivory by a crucifer specialist. Entomol Exp Appl 91:163–167 (1999).

[36] Brown PD and Morra MJ , Glucosinolate‐containing plant tissues as bioherbicides. J Agric Food Chem 43:3070–3074 (1995).

[37] Niemeyer HM , Hydroxamic acids derived from 2‐hydroxy‐2*H*‐1, 4‐benzoxazin‐3 (4*H*)‐one: key defense chemicals of cereals. J Agric Food Chem 3:1677–1696 (2009). 10.1021/jf8034034 19199602

[38] Villagrasa M , Guillamón M , Labandeira A , Taberner A , Eljarrat E and Barceló D , Benzoxazinoid allelochemicals in wheat: distribution among foliage, roots, and seeds. J Agric Food Chem 54:1009–1015 (2006). 16478210 10.1021/jf050898h

[39] Tsunoda T and van Dam NM , Root chemical traits and their roles in belowground biotic interactions. Pedobiologia 65:58–67 (2017).

[40] Huang Z , Haig T , Wu H , An M and Pratley JE , Correlation between phytotoxicity on annual ryegrass (*Lolium rigidum*) and production dynamics of allelochemicals within root exudates of an allelopathic wheat. J Chem Ecol 29:2263–2279 (2003). 14682511 10.1023/a:1026222414059

[41] Potter MJ , Vanstone VA , Davies KA , Kirkegaard J and Rathjen AJ , Reduced susceptibility of *Brassica napus* to *Pratylenchus neglectus* in plants with elevated root levels of 2‐phenylethyl glucosinolate. J Nematol 31:291–298 (1999). 19270899 PMC2620381

[42] von Rad U , Hüttl R , Lottspeich F , Gierl A and Frey M , Two glucosyltransferases are involved in detoxification of benzoxazinoids in maize. Plant J 28:633–642 (2001). 11851909 10.1046/j.1365-313x.2001.01161.x

[43] Knudsmark Jessing K , Duke SO and Cedergreen N , Potential ecological roles of artemisinin produced by *Artemisia annua* L. J Chem Ecol 40:100–117 (2014). 24500733 10.1007/s10886-014-0384-6

[44] Duke SO , Vaughn KC , Croom EM and Elsohly HN , Artemisinin, a constituent of annual wormwood (*Artemisia annua*). is a selective phytotoxin. Weed Sci 35:499–505 (2008).

[45] Wu H , Pratley JE , Lemerle D , An M and Liu DL , Autotoxicity of wheat (*Triticum aestivum* L.) as determined by laboratory bioassays. Plant and Soil 296:85–93 (2007).

[46] Chon SU and Kim JD , Biological activity and quantification of suspected allelochemicals from alfalfa plant parts. J Agron Crop Sci 188:281–285 (2002).

[47] Renne IJ , Sinn BT , Shook GW , Sedlacko DM , Dull JR , Villarreal D *et al*, Eavesdropping in plants: delayed germination via biochemical recognition. J Ecol 102:86–94 (2014).

[48] Chang X , Heene E , Qiao F and Nick P , The phytoalexin resveratrol regulates the initiation of hypersensitive cell death in *Vitis* cell. PLoS One 6:e26405 (2011). 22053190 10.1371/journal.pone.0026405 PMC3203900

[49] Duke SO , Cedergreen N , Belz RG and Velini ED , Hormesis: is it an important factor in herbicide use and allelopathy? Outlook Pest Manag 17:29–33 (2006).

[50] Sinkkonen A , Modelling the effect of autotoxicity on density‐dependent phytotoxicity. J Theor Biol 244:218–227 (2007). 16989866 10.1016/j.jtbi.2006.08.003

[51] Vilca Mallqui KS , Vieira JL , Guedes RNC and Gontijo LM , Azadirachtin‐induced hormesis mediating shift in fecundity‐longevity trade‐off in the Mexican bean weevil (Chrysomelidae: Bruchinae). J Econ Entomol 107:860–866 (2014). 24772571 10.1603/ec13526

[52] Kaur H , Kaur R , Kaur S , Baldwin IT and Inderjit S , Taking ecological function seriously: soil microbial communities can obviate allelopathic effects of released metabolites. PLoS One 4:e4700 (2009). 19277112 10.1371/journal.pone.0004700 PMC2650092

[53] Li Y‐P , Feng Y‐L , Chen Y‐J and Tian Y‐H , Soil microbes alleviate allelopathy of invasive plants. Sci Bull 60:1083–1091 (2015).

[54] Inderjit S and Nilsen ET , Bioassays and field studies for allelopathy in terrestrial plants: progress and problems. Crit Rev Plant Sci 22:221–238 (2003).

[55] Trezzi MM , Vidal RA , Balbinot Junior AA , von Hertwig Bittencourt H and da Silva Souza Filho AP , Allelopathy: driving mechanisms governing its activity in agriculture. J Plant Interact 11:53–60 (2016).

[56] Lankau RA , Resistance and recovery of soil microbial communities in the face of *Alliaria petiolata* invasions. New Phytol 189:536–548 (2011). 20958303 10.1111/j.1469-8137.2010.03481.x

[57] Smith CM and Chuang WP , Plant resistance to aphid feeding: behavioral, physiological, genetic and molecular cues regulate aphid host selection and feeding. Pest Manag Sci 70:528–540 (2014). 24282145 10.1002/ps.3689

[58] Wouters FC , Reichelt M , Glauser G , Bauer E , Erb M , Gershenzon J *et al*, Reglucosylation of the benzoxazinoid DIMBOA with inversion of stereochemical configuration is a detoxification strategy in lepidopteran herbivores. Angew Chemie ‐ Intl Ed 53:11320–11324 (2014). 10.1002/anie.201406643 25196135

[59] Baerson SR , Sánchez‐Moreiras AM , Pedrol‐Bonjoch N , Schulz M , Kagan IA , Agarwal AK *et al*, Detoxification and transcriptome response in *Arabidopsis* seedlings exposed to the allelochemical benzoxazolin‐2(3*H*)‐one. J Biol Chem 280:21867–21881 (2005). 15824099 10.1074/jbc.M500694200

[60] Chen S , Elzaki MEA , Ding C , Li ZF , Wang J , Z R‐s *et al*, Plant allelochemicals affect tolerance of polyphagous lepidopteran pest *Helicoverpa armigera* (Hübner) against insecticides. Pestic Biochem Physiol 154:32–38 (2019). 30765054 10.1016/j.pestbp.2018.12.009

[61] Hardy NB , Peterson DA , Ross L and Rosenheim JA , Does a plant‐eating insect's diet govern the evolution of insecticide resistance? Comparative tests of the pre‐adaptation hypothesis. Evol Appl 11:739–747 (2018). 29875815 10.1111/eva.12579 PMC5979754

[62] Kato‐Noguchi H and Peters RJ , The role of momilactones in rice allelopathy. J Chem Ecol 39:175–185 (2013). 23385366 10.1007/s10886-013-0236-9

[63] Cartwright DW , Langcake P , Pryce RJ , Leworthy DP and Ride JP , Isolation and characterization of two phytoalexins from rice as momilactones A and B. Phytochemistry 20:535–537 (1981).

[64] Batish DR , Singh HP , Kohli RK , Saxena DB and Kaur S , Allelopathic effects of parthenin against two weedy species, *Avena fatua* and *Bidens pilosa* . Environ Exp Bot 47:149–155 (2002).

[65] Datta S and Saxena DB , Pesticidal properties of parthenin (from *Parthenium hysterophorus*) and related compounds. Pest Manag Sci 57:95–101 (2001). 11455638 10.1002/1526-4998(200101)57:1<95::AID-PS248>3.0.CO;2-J

[66] Belz RG and Hurle K , Differential exudation of two benzoxazinoids ‐ one of the determining factors for seedling allelopathy of Triticeae species. J Agric Food Chem 53:250–261 (2005). 15656658 10.1021/jf048434r

[67] Macías FA , Marín D , Oliveros‐Bastidas A , Castellano D , Simonet AM and Molinillo JMG , Structure‐activity relationship (SAR) studies of benzoxazinones, their degradation products, and analogues. Phytotoxicity on problematic weeds *Avena fatua* L. and *Lolium rigidum* gaud. J Agric Food Chem 54:1040–1048 (2006). 16478215 10.1021/jf050903h

[68] Klun JA , Tipton CL and Brindley TA , 2,4‐dihydroxy‐7‐methoxy‐1,4‐benzoxazin‐3‐one (DIMBOA), an active agent in the resistance of maize to the European corn borer. J Econ Entomol 60:1529–1533 (1967).

[69] Gianoli E and Niemeyer HM , DIBOA in wild Poaceae: sources of resistance to the Russian wheat aphid (*Diuraphis noxia*) and the greenbug (*Schizaphis graminum*). Euphytica 102:317–321 (1998).

[70] Meyer SLF , Rice CP and Zasada IA , DIBOA: fate in soil and effects on root‐knot nematode egg numbers. Soil Biol Biochem 41:1555–1560 (2009).

[71] Virtanen AI , Hietala PK and Wahlroos Ö , Antimicrobial substances in cereals and fodder plants. Arch Biochem Biophys 69:486–500 (1957). 13445220 10.1016/0003-9861(57)90513-1

[72] Bravo HR and Lazo W , Antimicrobial activity of cereal hydroxamic acids and related compounds. Phytochemistry 33:569–571 (1993).

[73] Bertin C , Weston LA , Huang T , Jander G , Owens T , Meinwald J *et al*, Grass roots chemistry: *meta*‐tyrosine, an herbicidal nonprotein amino acid. Proc Natl Acad Sci U S A 104:16964–16969 (2007). 17940026 10.1073/pnas.0707198104 PMC2040483

[74] Gautam BK and Henderson G , Effects of *m*‐tyrosine on feeding and survival of Formosan subterranean termites (Isoptera: Rhinotermitidae). Ann Entomol Soc Am 101:1088–1093 (2008).

[75] Aronson JN and Wermus GR , Effects of *m*‐tyrosine on growth and sporulation of bacillus species. J Bacteriol 90:38–46 (1965). 16562040 10.1128/jb.90.1.38-46.1965 PMC315591

[76] Rietveld WJ , Allelopathic effects of juglone on germination and growth of several herbaceous and woody species. J Chem Ecol 9:295–308 (1983). 24407348 10.1007/BF00988047

[77] Thiboldeaux RL , Lindroth RL and Tracy JW , Differential toxicity of juglone (5‐hydroxy‐1,4‐naphthoquinone) and related naphthoquinones to saturniid moths. J Chem Ecol 20:1631–1641 (1994). 24242656 10.1007/BF02059885

[78] Clark AM , Jurgens TM and Hufford CD , Antimicrobial activity of juglone. Phytother Res 4:11–14 (1990).

[79] Pérez FJ , Allelopathic effect of hydroxamic acids from cereals on *Avena sativa* and *A. fatua* . Phytochemistry 29:773–776 (1990).

[80] Fomsgaard IS , Mortensen AG and Carlsen SCK , Microbial transformation products of benzoxazolinone and benzoxazinone allelochemicals ‐ a review. Chemosphere 54:1025–1038 (2004). 14664831 10.1016/j.chemosphere.2003.09.044

[81] Macías FA , Marín D , Oliveros‐Bastidas A , Castellano D , Simonet AM and Molinillo JMG , Structure‐activity relationships (SAR) studies of benzoxazinones, their degradation products and analogues. Phytotoxicity on standard target species (STS). J Agric Food Chem 53:538–548 (2005). 15686399 10.1021/jf0484071

[82] Macías FA , Chinchilla N , Varela RM , Oliveros‐Bastidas A , Marín D and Molinillo JMG , Structure‐activity relationship studies of benzoxazinones and related compounds. Phytotoxicity on *Echinochloa crus‐galli* (L.) P. Beauv. J Agric Food Chem 53:4373–4380 (2005). 15913298 10.1021/jf0502911

[83] Zhang S‐Z , Li Y‐H , Kong C‐H and Xu X‐H , Interference of allelopathic wheat with different weeds. Pest Manag Sci 72:172–178 (2016). 25641926 10.1002/ps.3985

[84] Bertholdsson N‐O , Early vigour and allelopathy ‐ two useful traits for enhanced barley and wheat competitiveness against weeds. Weed Res 45:94–102 (2005).

[85] Friebe A , Roth U , Kück P , Schnabl H and Schulz M , Effects of 2,4‐dihydroxy‐1,4‐benzoxazin‐3‐ones on the activity of plasma membrane H+‐ATPase. Phytochemistry 44:979–983 (1997).

[86] Sicker D , Frey M , Schulz M and Gierl A , Role of natural benzoxazinones in the survival strategy of plants. Int Rev Cytol 198:319–346 (2000). 10804466 10.1016/s0074-7696(00)98008-2

[87] Venturelli S , Petersen S , Langenecker T , Weigel D , Lauer UM and Becker C , Allelochemicals of the phenoxazinone class act at physiologically relevant concentrations. Plant Signal Behav 11:1–3 (2016). 10.1080/15592324.2016.1176818 PMC4973752 27088968

[88] Venturelli S , Belz RG , Kämper A , Berger A , von Horn K , Wegner A *et al*, Plants release precursors of histone deacetylase inhibitors to suppress growth of competitors. Plant Cell 27:3175–3189 (2015). 26530086 10.1105/tpc.15.00585 PMC4682303

[89] Corcuera LJ , Argandoña VH and Zúñiga GE , Allelochemicals in wheat and barley: role in plant‐insect interactions, in Allelopathy: Basics and Applied Aspects, ed. by SJH R and Rizvi V Chapman & Hall, London, pp. 119–127 (1992).

[90] Schulz M , Marocco A , Tabaglio V , Macias FA and Molinillo JMG , Benzoxazinoids in rye allelopathy ‐ from discovery to application in sustainable weed control and organic farming. J Chem Ecol 39:154–174 (2013). 23385365 10.1007/s10886-013-0235-x

[91] Movellan J , Rocher F , Chikh Z , Marivingt‐Mounir C , Bonnemain JL and Chollet JF , Synthesis and evaluation as biodegradable herbicides of halogenated analogs of L‐*meta*‐tyrosine. Environ Sci Pollut Res 21:4861–4870 (2014). 10.1007/s11356-012-1302-5 23224500

[92] Zer H , Mizrahi H , Malchenko N , Avin‐Wittenberg T , Klipcan L and Ostersetzer‐Biran O , The phytotoxicity of meta‐tyrosine is associated with altered phenylalanine metabolism and misincorporation of this non‐proteinogenic Phe‐analog to the plant's proteome. Front Plant Sci 11:1–18 (2020). 32210982 10.3389/fpls.2020.00140 PMC7069529

[93] Hejl AM , Einhellig FA and Rasmussen JA , Effects of juglone on growth, photosynthesis, and respiration. J Chem Ecol 19:559–568 (1993). 24248956 10.1007/BF00994325

[94] Hejl AM and Koster KL , Juglone disrupts root plasma membrane H+‐ATPase activity and impairs water uptake, root respiration, and growth in soybean (*Glycine max*) and corn (*Zea mays*). J Chem Ecol 30:453–471 (2004). 15112735 10.1023/b:joec.0000017988.20530.d5

[95] Willis RJ , *Juglans* spp., juglone and allelopathy. Allelopath J 7:1–55 (2000).

[96] Inderjit S and Duke SO , Ecophysiological aspects of allelopathy. Planta 217:529–539 (2003). 12811559 10.1007/s00425-003-1054-z

[97] Quader M , Daggard G , Barrow R , Walker S and Sutherland MW , Allelopathy, DIMBOA production and genetic variability in accessions of *Triticum speltoides* . J Chem Ecol 27:747–760 (2001). 11446298 10.1023/a:1010354019573

[98] Worthington M and Reberg‐Horton SC , Breeding cereal crops for enhanced weed suppression: optimizing allelopathy and competitive ability. J Chem Ecol 39:213–231 (2013). 23385368 10.1007/s10886-013-0247-6

[99] Gniazdowska A and Bogatek R , Allelopathic interactions between plants. Multi site action of allelochemicals. Acta Physiol Plant 27:395–407 (2005).

[100] Gressel J , Perspective: present pesticide discovery paradigms promote the evolution of resistance – learn from nature and prioritize multi‐target site inhibitor design. Pest Manag Sci 76:421–425 (2020). 31613036 10.1002/ps.5649

[101] Sparks TC , Hahn DR and Garizi NV , Natural products, their derivatives, mimics and synthetic equivalents: role in agrochemical discovery. Pest Manag Sci 73:700–715 (2017). 27739147 10.1002/ps.4458

[102] Regnault‐Roger C and Philogène BJR , Past and current prospects for the use of botanicals and plant allelochemicals in integrated pest management. Pharm Biol 46:41–52 (2008).

[103] Fomsgaard IS , Mortensen AG , Idinger J , Coja T and Blümel S , Transformation of benzoxazinones and derivatives and microbial activity in the test environment of soil ecotoxicological tests on *Poecilus cupreus* and *Folsomia candida* . J Agric Food Chem 54:1086–1092 (2006). 16478220 10.1021/jf050914a

[104] Fritz JI and Braun R , Ecotoxicological effects of benzoxazinone allelochemicals and their metabolites on aquatic nontarget organisms. J Agric Food Chem 54:1105–1110 (2006). 16478223 10.1021/jf050917n

[105] Lorsbach BA , Sparks TC , Cicchillo RM , Garizi NV , Hahn DR and Meyer KG , Natural products: a strategic lead generation approach in crop protection discovery. Pest Manag Sci 75:2301–2309 (2019). 30672097 10.1002/ps.5350

